# Proteomic Characterization of Cytoplasmic Lipid Droplets in Human Metastatic Breast Cancer Cells

**DOI:** 10.3389/fonc.2021.576326

**Published:** 2021-06-01

**Authors:** Alyssa S. Zembroski, Chaylen Andolino, Kimberly K. Buhman, Dorothy Teegarden

**Affiliations:** Department of Nutrition Science, Purdue University, West Lafayette, IN, United States

**Keywords:** breast cancer, metastasis, cytoplasmic lipid droplets, proteomics, triacylglycerol

## Abstract

One of the characteristic features of metastatic breast cancer is increased cellular storage of neutral lipid in cytoplasmic lipid droplets (CLDs). CLD accumulation is associated with increased cancer aggressiveness, suggesting CLDs contribute to metastasis. However, how CLDs contribute to metastasis is not clear. CLDs are composed of a neutral lipid core, a phospholipid monolayer, and associated proteins. Proteins that associate with CLDs regulate both cellular and CLD metabolism; however, the proteome of CLDs in metastatic breast cancer and how these proteins may contribute to breast cancer progression is unknown. Therefore, the purpose of this study was to identify the proteome and assess the characteristics of CLDs in the MCF10CA1a human metastatic breast cancer cell line. Utilizing shotgun proteomics, we identified over 1500 proteins involved in a variety of cellular processes in the isolated CLD fraction. Interestingly, unlike other cell lines such as adipocytes or enterocytes, the most enriched protein categories were involved in cellular processes outside of lipid metabolism. For example, cell-cell adhesion was the most enriched category of proteins identified, and many of these proteins have been implicated in breast cancer metastasis. In addition, we characterized CLD size and area in MCF10CA1a cells using transmission electron microscopy. Our results provide a hypothesis-generating list of potential players in breast cancer progression and offers a new perspective on the role of CLDs in cancer.

## Introduction

Breast cancer is the leading type of cancer among women in the United States and is predicted to account for 30% of new cancer cases in 2021 (1). Although breast cancer survival rates are relatively high if the cancer remains localized, life expectancy dramatically decreases once the cancer metastasizes to distant organs such as bone and lung (2). Therefore, understanding the characteristic features of metastatic breast cancer cells is critical in order to develop strategies to prevent the progression of breast cancer.

Metastatic breast cancer cells often exhibit altered lipid metabolism, which is an adaptation that allows cells to survive in nutrient-depleted conditions (3). One of these alterations includes the accumulation of neutral lipid in cytoplasmic lipid droplets (CLDs). The degree of CLD accumulation associates with breast cancer aggressiveness (4–6); however, the mechanisms behind this relationship are incompletely understood. CLDs are composed of a neutral lipid core of triacylglycerol (TAG) and/or cholesteryl esters surrounded by a phospholipid monolayer and associated proteins (7). The role of CLDs differs depending on cell type, for example serving as the body’s TAG storage pool in adipocytes (8), acting as a local energy source for skeletal and cardiomyocytes (9, 10), and mediating the process of dietary fat absorption in enterocytes (11). Although multiple hypotheses exist for how CLDs contribute to cancer progression, including protection from cellular stress or serving as a storage pool for fatty acids that can be used for cellular energy, biosynthetic processes, or signaling (6, 12, 13), the exact role of CLDs in metastatic breast cancer cells has not been determined.

Proteins that associate with CLDs serve a variety of functions, but their role in metastasis in unknown. A common function of CLD proteins is to mediate TAG synthesis and lipolysis, reflecting the main purpose of CLDs in storing neutral lipid and maintaining cellular lipid homeostasis (14). However, recent functional studies of CLD proteins demonstrate novel cellular roles for CLDs by regulating cellular protein location, degradation, and functional activity. For example, histone proteins and transcription factors sequester at the CLD as a mechanism to regulate gene expression (15–17). In addition, some CLD proteins are destined for degradation (18) such as apolipoprotein B-100, a component of very-low-density lipoproteins, which translocates from the endoplasmic reticulum (ER) to the CLD for degradation in hepatocytes (19, 20). Finally, CLD proteins may also have specific functions on the CLD, for example mediating inflammatory signaling pathways (21–23). Despite the identification of proteins involved in a variety of roles in CLD proteomic studies, the functional significance of the majority of CLD proteins has yet to be uncovered. Further, the functional significance of CLD proteins in metastatic breast cancer cells and whether they reflect unique roles for CLDs in cancer is unknown.

The purpose of this study was to identify the proteome of CLDs in metastatic breast cancer cells to generate hypotheses about how CLDs promote breast cancer progression and contribute to altered lipid metabolism and/or other cell functions. To do this, we performed untargeted shotgun proteomic analysis and utilized transmission electron microscopy (TEM) to identify the proteome and characteristics of CLDs from the human metastatic breast cancer cell line, MCF10CA1a.

## Materials and Methods

### Cell Culture

The MCF10CA1a human metastatic mammary cell line was utilized for these studies. Cells were cultured in Dulbecco’s Modified Eagle Medium: Nutrient Mixture F-12 (DMEM/F12, 1:1), supplemented with 5% horse serum, 100 units/mL penicillin, and 100 µg/mL streptomycin in a humidified environment at 37°C with 5% CO_2_. Cells were harvested at 70-80% confluence for each experiment.

### CLD Isolation

Cells from eight 150 mm dishes were pooled and considered one sample. Four samples were prepared for CLD isolation as follows. Cells were rinsed with phosphate buffered saline (PBS, pH 7.4, 137 mM NaCl, 2.7 mM KCl, 8 mM Na_2_HPO_4_, and 2 mM KH_2_PO_4_) scraped and pelleted by centrifugation. CLDs were isolated from pelleted cells using a previously established sucrose gradient ultracentrifugation protocol (24, 25). Briefly, cells were lysed in ice cold sucrose lysis buffer (175 mM sucrose, 10 mM HEPES and 1 mM EDTA pH 7.4) and disrupted by passing through a 23 gauge, 1 inch needle. An aliquot was taken representing the whole cell lysate (WCL) to be used for later applications. The remaining lysate was transferred into a 13.2 mL Open-Top Thinwall UltraClear tube (Beckman Coulter, #344059) and ice-cold lysis buffer was layered on top of the lysate. Samples were centrifuged at 100,000 x g at 4°C for one hour. After centrifugation, the white floating fraction (FF) from each sample was aspirated using a pipette. The remaining soluble and pellet fractions were removed in 1 mL increments. Samples were stored at -80°C until analysis.

### Triacylglycerol and Protein Concentration

TAG concentrations of each fraction were measured using the Wako L-Type Triglyceride M kit (FUJIFILM Wako Diagnostics U.S.A.). Protein concentrations of each fraction were measured using the Pierce™ BCA Protein Assay Kit (Thermo Fisher Scientific).

### Validation of CLD Isolation by Western Blotting

An aliquot of each isolated fraction (CLD fraction through pellet fraction) and the WCL was delipidated with 2:1 chloroform:methanol, then proteins were precipitated with ice-cold acetone. The precipitated proteins were pelleted by centrifugation, then dried and resuspended in Laemmli loading buffer. Samples were subjected to SDS-PAGE using a 12% Tris-glycine gel (Bio-Rad #4561046). Samples were loaded into the gel by volume: 10 µL each of the FF through fraction 10, 5 µL of the pellet fraction and 5 µL of the WCL. See **Supplementary Figure 1** for representative Ponceau stain demonstrating differences in protein levels between fractions. The membrane was probed with one of the following primary antibodies at a 1:1,000 concentration (PLIN3, Sigma-Aldrich HPA006427; GAPDH, Cell Signaling Technologies #14C10; CANX, Santa Cruz Biotechnology SC-11397). After washing, a fluorescent secondary antibody was added at a concentration of 1:10,000 (LI-COR IRDye donkey anti-rabbit 680RD, 926-68073). Membranes were imaged using the LI-COR Odyssey CLx Imaging System (LI-COR Biosciences).

### Transmission Electron Microscopy

One 60 mm dish of MCF10CA1a cells and one 60 mm dish of MCF10A-*ras* cells were prepared for TEM. Cells were fixed in 2.5% glutaraldehyde in 0.1 M sodium cacodylate buffer, rinsed, and embedded in agarose. Small pieces of cell pellet were post-fixed in 1% osmium tetroxide containing 0.8% potassium ferricyanide and stained in 1% uranyl acetate. They were then dehydrated with a graded series of ethanol, transferred into acetonitrile, and embedded in EMbed-812 resin. Thin sections were cut on a Reichert-Jung Ultracut E ultramicrotome and post-stained with 4% uranyl acetate and lead citrate. Images were acquired on a FEI Tecnai T12 electron microscope equipped with a tungsten source and operating at 80kV.

### CLD Size Analysis

Acquired TEM images were analyzed using ImageJ (26). 50 cells were counted and used for CLD analysis. CLD diameter was assessed using ImageJ.

### Immunocytochemistry

MCF10CA1a cells were cultured in a #1.5H-N high performance glass bottom 12 well plate (Cellvis) and processed for immunofluorescence microscopy. The cells were fixed in 4% paraformaldehyde, permeabilized with 0.1% Triton X-100, and blocked with BlockAid (Invitrogen, B10710). Cells were probed with antibodies for PLIN3, SQLE, and NSDHL (Sigma, HPA006427; SantaCruz Biotechnologies, sc-271651; Atlas Antibodies, HPA000571, respectively). Proteins were detected using secondary AlexaFluor antibodies (Life Technologies, A-21070 and A-21052), and cells were counterstained for neutral lipids using 1 μg/mL 4,4-difluoro-1,3,5,7,8-pentamethyl-4-bora-3a,4a-diaza-s-indacene (BODIPY 493/503; Life Technologies, Grand Island, NY, United States), and for nuclei using 300 nM 4’,6-Diamidino-2-Phenylindole, Dihydrochloride (DAPI; Invitrogen, D1306). Samples were imaged using a Nikon A1R-MP inverted confocal microscope (Nikon Instruments Inc., Melville, NY, United States). Images were acquired using the Plan Apo λ 100x Oil objective, 76.63 µm pinhole size, and DAPI, FITC, and Cy5 lasers. All image processing was conducted using Nikon NIS-Elements AR acquisition and analysis software. A Landweber 2D deconvolution algorithm was implemented, with point scan confocal modality, clear noise, and 12, 12, 12 iterations.

### CLD Protein Isolation and In-Solution Digestion

An aliquot of each CLD fraction containing 50 μg protein was prepared for proteomic analysis. The CLD fractions were delipidated and precipitated as above. The dried protein pellets were reduced and solubilized using 10 mM dithiothreitol/8 M urea, then alkylated using iodoethanol. Samples were dried using a vacuum centrifuge. Proteins were digested with 4 μg Trypsin/Lys-C Mix, Mass Spec Grade (Promega) per sample using a barocycler at 50°C, 20 kpsi, 60 cycles (Barocycler NEP2320, Pressure Biosciences, INC). Peptides were cleaned with MacroSpin C18 spin columns (The Nest Group, Inc) and dried using a vacuum centrifuge. Dried peptides were resuspended in 3% acetonitrile/0.1% formic acid in preparation for mass spectrometry.

### Liquid Chromatography/Tandem Mass Spectrometry (LC-MS/MS)

Samples were analyzed by reverse-phase LC-ESI-MS/MS system using the Dionex UltiMate 3000 RSLC nano System coupled to the Orbitrap Fusion Lumos Mass Spectrometer (Thermo Fisher Scientific). Peptides were loaded onto a trap column (300 mm ID ´ 5 mm) packed with 5 mm 100 Å PepMap C18 medium, then separated on a reverse phase column (50-cm long × 75 µm ID) packed with 2 µm 100 Å PepMap C18 silica (Thermo Fisher Scientific). The column temperature was maintained at 50°C. All MS measurements were performed in positive ion mode using a 120 minute LC gradient and standard data-dependent mode. MS data were acquired with a Top20 data-dependent MS/MS scan method.

### LC-MS/MS Data Analysis

LC-MS/MS data were analyzed using MaxQuant software version 1.6.3.4 (27–29). Data was searched against the UniProtKB *Homo sapiens* reference proteome (www.uniprot.org). Trypsin/P and Lys-C were selected with a maximum of 2 missed cleavages. Oxidation of methionine was set as a variable modification, iodoethanol set as a fixed modification. First search peptide mass tolerance was set to 20 ppm, main search peptide mass tolerance was set to 10 ppm. False discovery rate was set to 1%. Match between runs was selected and Label-free quantification (LFQ) was used.

### Proteomic Data Analysis

Reverse identifications and contaminants were removed from the dataset. LFQ values were subjected to Log2 transformation. A protein was considered identified if it was present in at least three out of four samples. Uniprot accession numbers in the Majority Protein IDs column were used to categorize proteins into Gene Ontology Biological Process (GO_BP) terms using The Database for Annotation, Visualization and Integrated Discovery (DAVID) v6.8 (30, 31). Functional relationships between proteins were visualized using STRING version 11 (32).

## Results

### Characterization of CLDs in MCF10CA1a Cells

**Figure 1:**
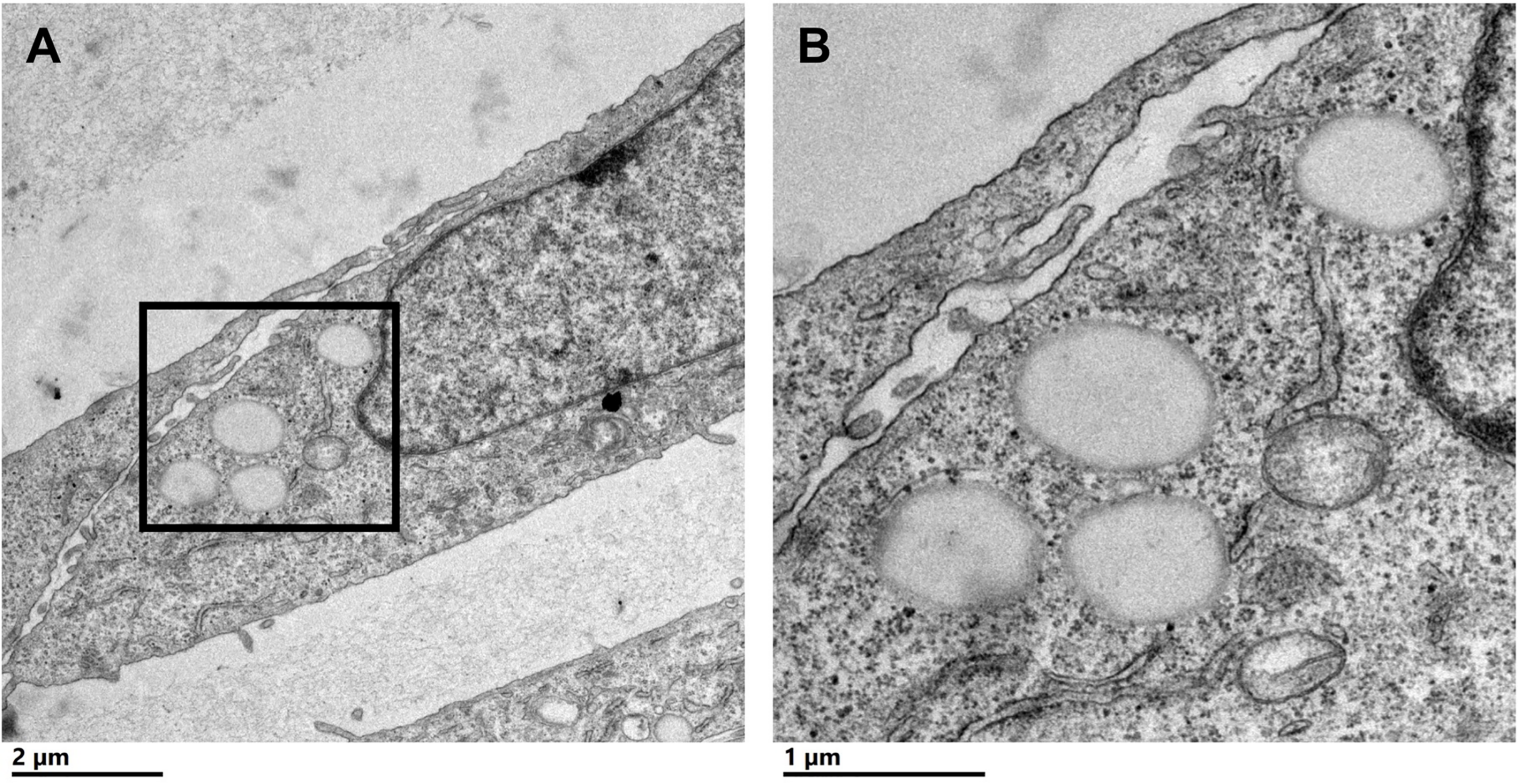
Cytoplasmic lipid droplets (CLDs) are present in MCF10CA1a cells. **(A)** Representative transmission electron microscopy (TEM) image of a MCF10CA1a cell containing CLDs (boxed region), scale bar 2 μm. **(B)** Magnified image of the CLDs present in **(A)** scale bar 1 μm.

**Figure 2:**
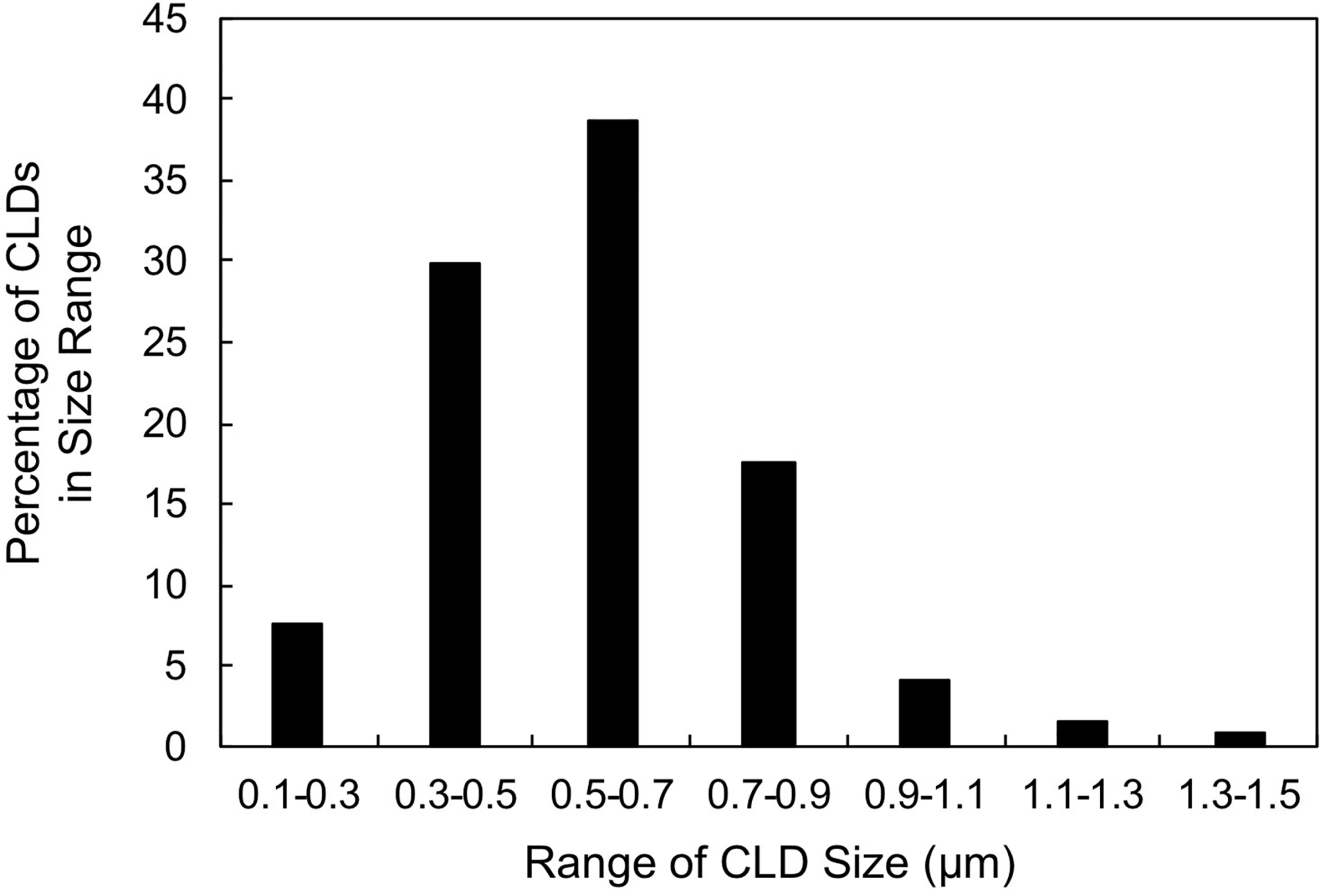
Cytoplasmic lipid droplet (CLD) size distribution. Percentage of CLDs analyzed in **Table 1** within the indicated size range. 50 cells were counted and used for the analysis. CLD diameter was measured using ImageJ.

To characterize CLDs in the metastatic breast cancer MCF10CA1a cell line, we visualized cells by TEM. A representative MCF10CA1a cell containing CLDs is shown in **Figure 1A**. CLDs present within the cell are highlighted (**Figure 1B**). To determine the distribution of CLDs across cells, we assessed the number and diameter of CLDs per cell (**Table 1** and **Figure 2**). Ninety percent of cells counted contained CLDs, and the number of CLDs per cell ranged from 0-41. CLD diameter also varied across cells. CLD diameter ranged from 0.17-1.38 μm (**Figure 2**), with an average CLD diameter of 0.58 µm. As expected, only 10% of non-metastatic MCF10A-*ras* cells of the same cell series analyzed contained CLDs (data not shown). A representative MCF10A-*ras* cell without CLDs is shown in **Supplementary Figure 2**. Due to the absence of CLDs in most MCF10A-*ras* cells, we were unable to isolate CLDs from MCF10A-ras cells and therefore only assessed the proteome of CLDs from MCF10CA1a cells.

**Table 1:** Number and size of CLDs within MCF10CA1a cells. 50 cells were counted and used for the analysis.

% of cells containing CLDs	# CLDs per cell	Average # CLDs per cell	CLD diameter range (μm)	Average CLD diameter (μm)
90	0-41	12	0.17-1.38	0.58

CLD diameter was measured using ImageJ. Distribution of CLD diameters is shown in **Figure 2**.

### CLD Isolation From MCF10CA1a Cells

**Figure 3:**
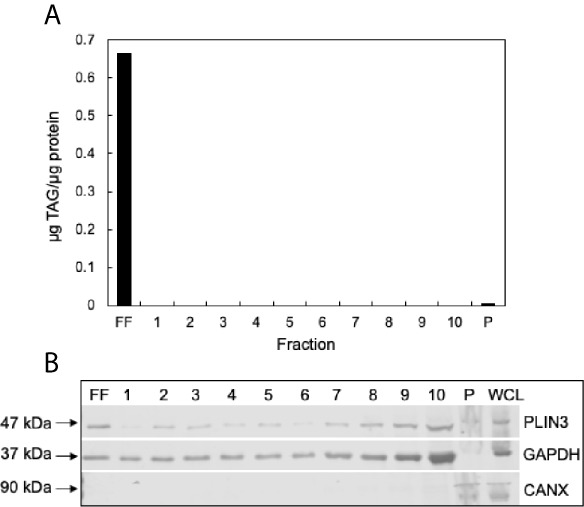
Validation of cytoplasmic lipid droplet (CLD) isolation. **(A)** Triacylglycerol (TAG) to protein ratio of each isolated fraction. CLDs were isolated from MCF10CA1a cells using sucrose density gradient ultracentrifugation. Fractions were removed sequentially from the top of the gradient to the bottom. Floating fraction (FF): isolated CLDs, 1-10: soluble fractions, P: pellet. **(B)** Western blot of isolated fractions and whole cell lysate (WCL). Fractions were loaded by volume: 10 μL FF-10, 5 μL P and WCL. Membrane was probed for markers of CLDs (PLIN3), cytosol (GAPDH), and endoplasmic reticulum (CANX). Approximate molecular weight markers for each protein are listed. See **Supplementary Figure 1** for a representative Ponceau stain reflecting the relative levels of protein in each fraction.

**Figure 4:**
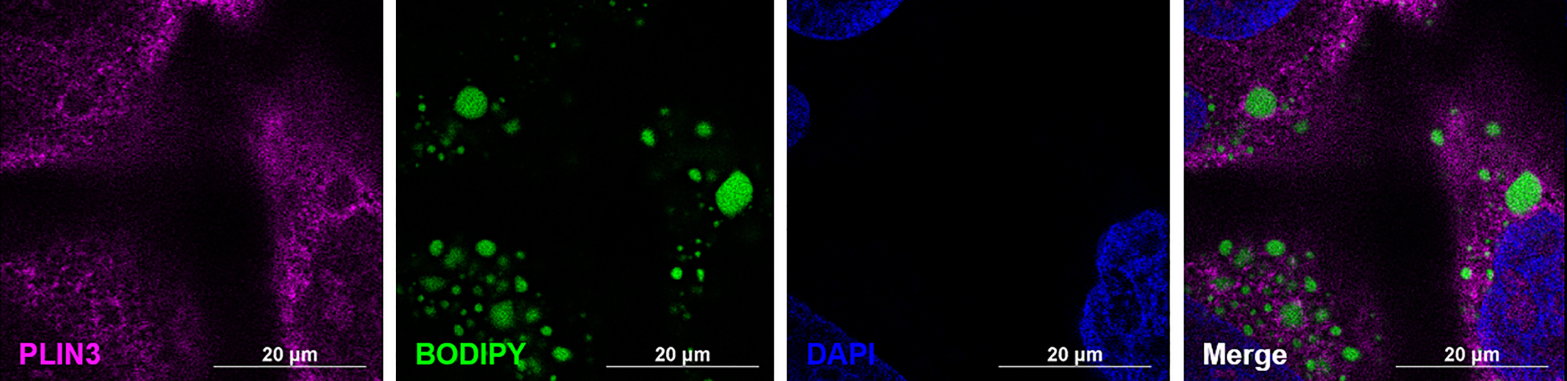
PLIN3 surrounds cytoplasmic lipid droplets (CLDs) in MCF10CA1a cells. Representative immunofluorescence images of MCF10CA1a cells. Cells were stained with Alexa Fluor 633 to visualize PLIN3, BODIPY to visualize CLDs, and DAPI to visualize nuclei. Signals from all three channels were merged for the final image.

To confirm successful isolation of CLDs from MCF10CA1a cells, we determined the TAG to protein ratio of each isolated fraction after sucrose density gradient ultracentrifugation (**Figure 3A**). A high TAG to protein ratio in the floating fraction (FF) indicates the presence of CLDs. In addition, we determined the purity of our isolation based on the presence of specific cell component markers in each isolated fraction (**Figure 3B**). Perilipin (PLIN) 3, a bona-fide CLD-associated protein and marker of CLDs (33), is present in the FF. PLIN3 resides in the cytosol but associates with CLDs when CLDs are present (34), which is consistent with its identification in the soluble fractions. The localization of PLIN3 to CLDs was confirmed by immunocytochemistry (**Figure 4**). Glyceraldehyde 3-phosphate dehydrogenase (GAPDH), a cytosolic marker, is present in the FF and soluble fractions but absent in the pellet fraction (**Figure 3B**). GAPDH is identified in CLD proteomic studies of certain cell types (35, 36), and the identification of GAPDH in the FF suggests GAPDH is a CLD-associated protein in MCF10CA1a cells. Calnexin (CANX), a marker of ER, is present in only the pellet fraction (**Figure 3B**), as expected based on published CLD isolation protocols (24). Isolated fractions were loaded by volume and therefore contain different amounts of protein; see representative Ponceau stain in **Supplementary Figure 1** for the relative amount of protein in each fraction.

### Proteomic Characterization of CLDs in MCF10CA1a Cells

**Figure 5:**
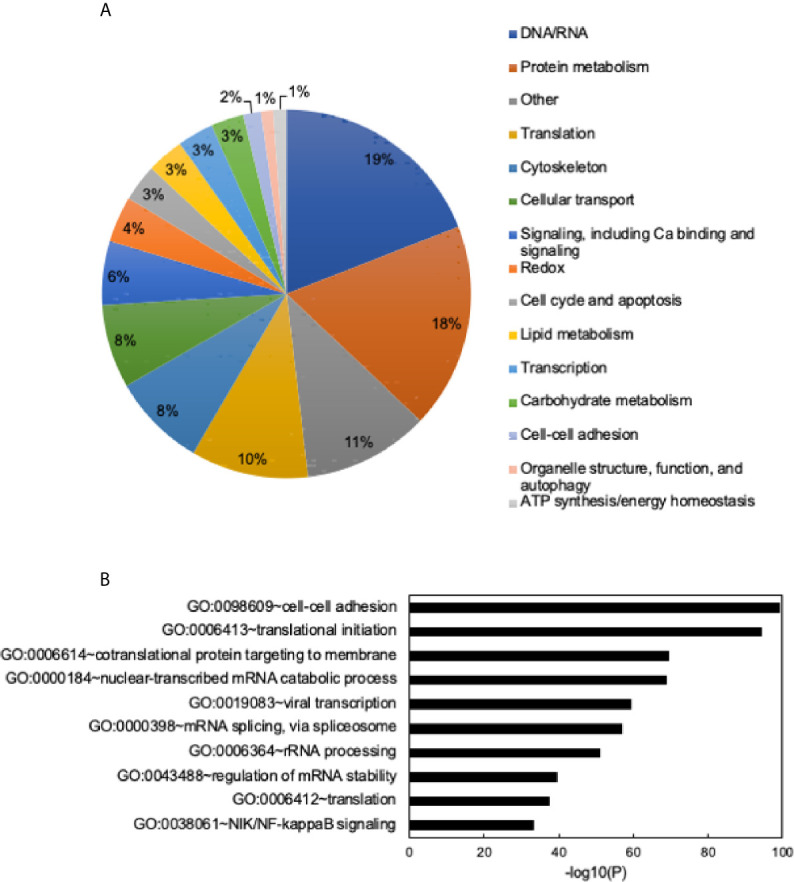
General functions of identified proteins and Gene Ontology (GO) term enrichment. **(A)** Identified proteins grouped into general categories. Data shown as a percent of total proteins identified. Categories with the highest to lowest percent of proteins listed from top to bottom and are read clockwise around the pie chart. **(B)** Chart of the top 10 most enriched Gene Ontology Biological Process (GO_BP) terms. Most to least enriched term listed from top to bottom. Data shown as -log10 (p-value). Enrichment scores/p-values calculated in DAVID. See **Supplementary Table 2** for full list of enriched GO terms and enrichment scores.

**Figure 6:**
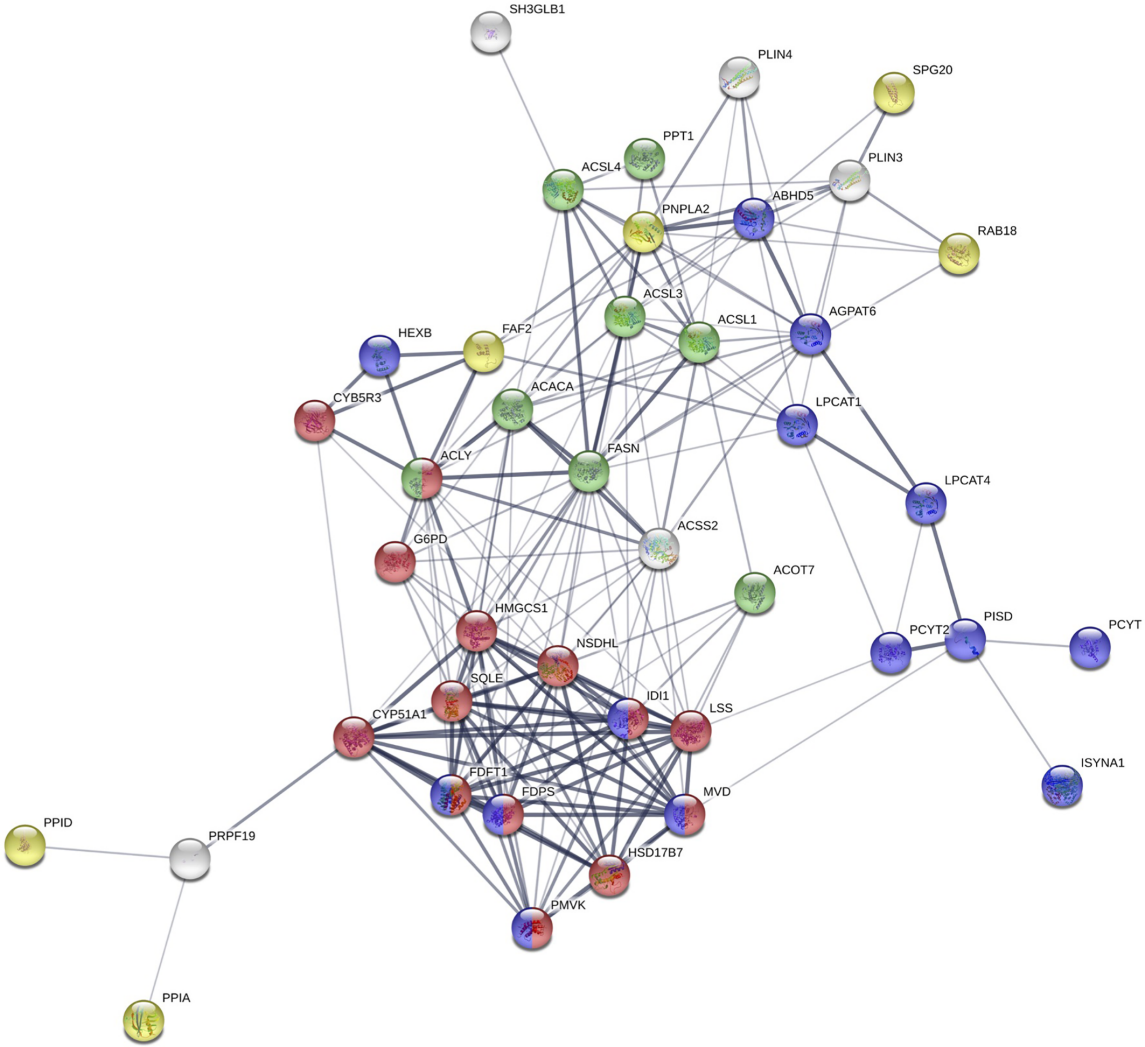
STRING analysis of identified proteins involved in lipid metabolism. Proteins with known functions in lipid metabolism and those associated with lipid-related Gene Ontology Biological Process (GO_BP) terms. Red: cholesterol biosynthetic process; green: fatty-acyl-CoA metabolic process; purple: phospholipid metabolic process; yellow: lipid droplet organization.

**Figure 7:**
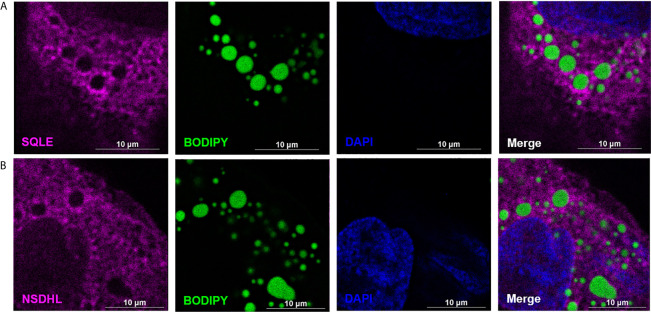
SQLE and NSDHL localize to cytoplasmic lipid droplets (CLDs) in MCF10CA1a cells. Representative immunofluorescence images of MCF10CA1a cells. Cells were stained with Alexa Fluor 633 to visualize SQLE **(A)** and NSDHL **(B)**, BODIPY to visualize CLDs, and DAPI to visualize nuclei. Signals from all three channels were merged for the final image in **(A, B)**.

To determine the proteome of CLDs in MCF10CA1a cells, we performed untargeted shotgun proteomic analysis of the isolated CLD fraction using LC-MS/MS. We identified 1534 proteins (**Supplementary Table 1**) that are involved in a wide array of cellular functions (**Figure 5A**). Many of the proteins identified have functions in DNA and RNA metabolic processes (19%) and protein metabolism (18%). To determine whether a specific category of proteins was overrepresented in our dataset, we sorted proteins by Gene Ontology Biological Process (GO_BP) enrichment (**Figure 5B**). Cell-cell adhesion was the most enriched category of proteins identified, followed by translational initiation, and cotranslational protein targeting to membrane (**Figure 5B** and **Supplementary Table 2**). Surprisingly, proteins involved in lipid metabolism comprise only 3% of the proteins identified (**Figure 5A**), and lipid metabolic terms are not represented within the top 50 most enriched GO_BP categories (**Supplementary Table 2**). Low abundance of lipid metabolism proteins is in contrast to other CLD proteomic studies, where they are frequently enriched (14). We analyzed the 41 proteins identified as associated with lipid metabolism (**Figure 6**). Most of these proteins are involved in cholesterol synthesis, including hydroxymethylglutaryl-CoA synthase, cytoplasmic (HMGCS1), squalene monooxygenase (SQLE), and sterol-4-alpha-carboxylate 3-dehydrogenase, decarboxylating (NSDHL). The localization of SQLE and NSDHL to CLDs was confirmed by immunocytochemistry (**Figure 7**). Both SQLE and NSDHL are shown to concentrate around CLDs. Other identified proteins have basic roles in CLD metabolism, including lipolysis [patatin-like phospholipase domain-containing protein 2/adipose triglyceride lipase (PNPLA2/ATGL), 1-acylglycerol-3-phosphate O-acyltransferase ABHD5 (ABHD5)], phospholipid synthesis [choline-phosphate cytidylyltransferase A (PCYT1A)], TAG synthesis [glycerol-3-phosphate acyltransferase 4 (GPAT4)], and the PLINs (PLIN3 and PLIN4).

### Proteins Involved in Cell-Cell Adhesion Are Implicated in Breast Cancer Progression

We further analyzed the proteins belonging to the cell-cell adhesion category, as this was the most enriched GO_BP term of proteins identified (**Figure 5B**). To determine how CLDs and their proteins contribute to breast cancer metastasis, we chose proteins in the cell-cell adhesion category that also had GO_BP terms in cell migration and signaling. Proteins with these criteria are listed in **Table 2**. Many of these proteins have been shown to promote breast cancer progression, and the references for each are included in **Table 2**.

**Table 2:** Proteins in cell-cell adhesion are associated with breast cancer metastasis.

Protein name	Gene	General function	Proposed role in breast cancer metastasis	References
IQ motif containing GTPase activating protein 1	IQGAP1	Scaffold protein; signaling and cytoskeleton dynamics	Promotes cell proliferation, migration, tumor growth	(37–41)
Serine/threonine kinase 24	STK24	MAPK signaling	Promotes cell proliferation, tumor growth	(42)
S100 calcium binding protein P	S100P	Calcium signaling	Promotes cell proliferation, migration, motility	(43–45)
Fascin actin-bundling protein 1	FSCN1	Actin-binding protein; cell adhesion, motility, migration	Promotes metastasis through NFκB and STAT3 signaling	(46–51)
GIPC PDZ domain containing family member 1	GIPC1	Scaffold protein; signaling	Involved in cell cycle, apoptosis, motility	(52, 53)
Profilin 1	PFN1	Actin-binding protein; cytoskeletal dynamics	Suppresses cell migration and cell cycle	(54–57)
Tumor-associated calcium signal transducer 2	TACSTD2	Calcium signaling	Promotes cell growth, migration, proliferation through AKT signaling	(58, 59)
Syndecan binding protein	SDCBP	Adaptor protein; signaling and cytoskeletal dynamics	Promotes cell proliferation, growth, motility, cell cycle	(60–63)
RAB1A, member RAS oncogene family	RAB1A	Vesicle trafficking from ER to Golgi	Involved in cell proliferation, migration, EMT; involved in mTORC1 signaling	(64, 65)
STE20 like kinase	SLK	Apoptosis, cytoskeletal dynamics	Promotes cell migration	(66)
Coronin 1B	CORO1B	Actin-binding protein; cell motility	Involved in cell cycle progression	(67)
Heat shock protein family A (Hsp70) member 5	HSPA5	Protein folding	Promotes cell motility and proliferation	(68–70)
Microtubule associated protein RP/EB family member 1	MAPRE1	Microtubule dynamics	Promotes cell proliferation and tumor growth	(71)
Radixin	RDX	Binds actin	Involved in cell motility; interacts with ERBB2 receptors	(72, 73)
Signal transducer and activator of transcription 1	STAT1	Transcription factor; responds to cytokines and growth factors	Either promotes or inhibits tumor growth	(74–77)

List of proteins within the Gene Ontology Biological Process (GO_BP) category “cell-cell adhesion” that also have GO_BP terms of signaling and/or cell migration.

## Discussion

To determine mechanisms by which CLDs contribute to breast cancer metastasis, we examined the characteristics and proteome of CLDs in the human metastatic breast cancer cell line, MCF10CA1a, using TEM and LC-MS/MS. We found that the majority of MCF10CA1a cells analyzed contain multiple CLDs that associate with a variety of proteins. To our knowledge, this is the first report of the proteome of CLDs in metastatic breast cancer cells. We identified 1534 proteins in the isolated CLD fraction representing a wide array of cellular functions. Many of the proteins identified are implicated in breast cancer metastasis. Our results provide a hypothesis-generating list of potential players contributing to cancer progression and provide a new perspective on the role of CLDs in metastatic breast cancer.

Our results are consistent with previous work demonstrating that neutral lipid accumulation in breast cancer cells correlates with cancer aggressiveness (4, 5, 78–80). MCF10CA1a cells are the most metastatic in the MCF10A series of breast cancer progression (81) and contain twelve times more TAG than the non-metastatic MCF10A-*ras* cell line from which they were derived (82). Consistently, while most MCF10CA1a cells analyzed contained at least one CLD (**Table 1**), almost no CLDs were present in non-metastatic MCF10A-*ras* cells (**Supplementary Figure 2**). The underlying mechanism driving increased CLDs in metastatic MCF10CA1a cells and not in MCF10A-*ras* cells is not clear, however, several factors may contribute. For example, metastatic breast cancer cells may have an increased ability, compared to non-metastatic cells, to take up or synthesize FA and cholesterol which are used as substrates for TAG and cholesteryl ester synthesis and subsequently stored in CLDs (6). Overall, these results support our use of the MCF10CA1a cell line as a model of mammary metastasis to investigate the CLD proteome.

CLD size is often used to estimate the amount of cellular neutral lipid storage and the metabolic state of the cell. For example, cells that store large amounts of TAG, such as adipocytes (83) and enterocytes (84), have large CLDs (ranging up to 100 µm), whereas other cell types tend to have smaller CLDs. Consistent with the size of CLDs in cell types that do not store large amounts of TAG, including skeletal myocytes (85), hepatocytes (86), and Chinese Hamster Ovary (CHO) cells (87), the diameter of CLDs in the MCF10CA1a cells averaged 0.58 µm (**Table 1**). Further, the distribution of CLDs of various sizes in MCF10CA1a cells (**Figure 2**) may reflect different pools of CLDs that have potentially distinct functions (35, 88). For example, specific pools of CLDs in brown adipose tissue are differentially involved in fatty acid oxidation or TAG synthesis (89). It is possible that unique pools of CLDs with different functions may exist in MCF10CA1a cells; however, this requires further investigation.

The proteome of CLDs identified in MCF10CA1a cells has similarities and differences compared to that of other cell types. Many of the proteins identified are consistent with the general categories of proteins commonly found on CLDs. These include proteins involved in lipid and CLD metabolism, translation, protein folding and degradation, cytoskeleton, and histones (14). Several of the proteins identified involved in lipid metabolism have been validated as CLD-associated proteins and also have functional roles at the CLD surface, including PLIN3 in CLD maintenance (90), GPAT4 (91) and PCYT1A (92, 93) in CLD expansion and size, ATGL in CLD lipolysis (94), and NSDHL in cholesterol synthesis (95, 96). The identification of lipid metabolism proteins on CLDs in MCF10CA1a cells suggests CLDs across cell types may share similar lipid metabolic machinery and core CLD proteins.

A key difference between the proteome of CLDs in MCF10CA1a cells and that of other cell types is the representation of proteins in the commonly identified categories. For example, lipid metabolism was not a highly enriched protein category in MCF10CA1a cells as it is in other cell types (14). Further, many of the proteins we identified in the lipid metabolism category are involved in cholesterol metabolism, suggesting CLDs in MCF10CA1a cells may store cholesterol (97, 98). Consistently, cholesteryl ester accumulation and altered cholesterol metabolism is a common feature of cancer (99, 100). We found that two enzymes involved in cholesterol synthesis, NSDHL and SQLE, concentrate in areas around CLDs in MCF10CA1a cells (**Figure 7**).

The identification of NSDHL with CLDs in breast cancer cells is consistent with previous observations of its functional association with CLDs and role in metastasis. NSDHL modifies lanosterol before its synthesis into cholesterol (101), and has been shown to localize to CLDs upon oleate loading in CHO cells (95) and in COS-7 cells (96). In fact, oleate loading and CLD formation in CHO cells decreased the synthesis of C-27 sterols, which includes cholesterol, and increased the synthesis of precursor sterols, including lanosterol (95). These results suggest the localization of NSDHL to CLDs may be a mechanism to regulate cholesterol synthesis. NSDHL has also been shown to promote breast cancer progression. NSDHL is present at higher protein levels in metastatic compared to non-metastatic breast cancer cell lines (102), and knockdown of NSDHL in metastatic BT-20 and MDA-MB-231 cells reduced cell viability, colony formation, and cell migration (102). However, whether these effects are due to lack of NSDHL itself or lack of cholesterol synthesis due to NSDHL inhibition is unclear. Thus, the localization of NSDHL to CLDs in MCF10CA1a cells shown in this study suggests that it may promote breast cancer progression by regulating cholesterol synthesis. Future studies are required to determine the role of NSDHL on CLDs in MCF10CA1a cells and its contribution to metastasis.

The identification of SQLE with CLDs in breast cancer cells is also consistent with previous observations of its functional association with CLDs and role in metastasis. SQLE catalyzes the epoxidation of squalene and is considered the second rate-limiting step in cholesterol synthesis (103). SQLE localizes to CLDs in yeast cells (104) and has been shown to regulate CLD dynamics. For example, inhibition of SQLE results in CLD clustering and squalene accumulation in yeast (105), and CLD accumulation in MCF7 breast cancer cells (106). SQLE may regulate CLD dynamics by interacting with microprotein CASIMO1 (106). CASIMO1 in MCF7 cells was shown to regulate the expression of SQLE as well as CLD formation. How CASIMO1 and/or SQLE influences CLDs is not clear; however, it may involve changes in the cytoskeleton. Interestingly, SQLE has been identified as an oncogene in breast cancer cells (107), suggesting it plays a role in breast cancer metabolism. Consistently, inhibiting SQLE in MCF7 breast cancer cells reduces cell proliferation and ERK phosphorylation/activation (106), which is a key factor involved in initiating cell proliferation and migration in cancer cells (108). ERK phosphorylation and activation has previously been shown to be regulated by SQLE in other cell types including hepatocellular carcinoma cells (109) and lung squamous cell carcinoma cells (110). In fact, SQLE-mediated cholesterol synthesis preserves breast cancer stem cell stemness through PI3K/AKT signaling, another proliferative survival pathway, upon stabilization of SQLE mRNA by long non-coding RNA 030 and poly(rC) binding protein 2 (111). Therefore, the metabolites produced by the action of SQLE may activate cell signaling pathways necessary for cancer cell proliferation. Overall, these results suggest that the localization of enzymes involved in cholesterol synthesis to CLDs in MCF10CA1a cells may be a metabolic adaptation by cancer cells that stimulates cell proliferation. Future studies are required to determine the role of SQLE on CLDs in MCF10CA1a cells.

Instead of lipid metabolism proteins representing the majority of the CLD proteome, proteins with roles in cell-cell adhesion, translation, and mRNA metabolism were the most prevalent in the CLD fraction of MCF10CA1a cells, suggesting these proteins may have a novel functional role on CLDs in cancer. The most enriched category of proteins identified were those involved in cell-cell adhesion. This is particularly interesting, since loss of cell adhesion is a critical first step in the metastatic cascade (112). Many of the proteins identified in this category have been implicated in breast cancer metastasis (**Table 2**), suggesting CLDs may play a novel role in this process. For example, CLDs may serve as a hub for signaling pathways and cytoskeletal remodeling proteins that are needed to facilitate the epithelial-mesenchymal transition (EMT). However, CLD proteins may either play an active role at the CLD surface or may be mislocalized from their typical cell location, which could interrupt their function and contribute to metastasis. Future studies are required to determine the role of signaling and cytoskeletal proteins identified in **Table 2** on CLDs in MCF10CA1a cells.

Another category of proteins identified in the isolated CLD fraction of MCF10CA1a cells is RNA binding proteins and translational proteins. Some of these proteins are also implicated in cell motility and breast cancer metastasis (113, 114), suggesting their localization on CLDs contributes to metastatic potential. For example, downregulation of the RNA-binding protein ZBP1 in metastatic breast cancer cells increased cell migration by altering the expression of mRNAs involved in cell-cell adhesion, cytoskeleton, and cell proliferation (115). In addition, overexpression of the 60S ribosomal subunit RPL15 in circulating tumor cells isolated from patients with metastatic breast cancer increased the translation of ribosomal proteins and proteins involved in cell proliferation, and when injected into mice resulted in increased metastasis and tumor formation (116). Interestingly, RNA localizes to CLDs in human mast cells (117) and ribosomes localize to CLDs in human monocyte U937 cells and leukocytes (118). It is possible that CLDs in MCF10CA1a cells house RNA-binding and translational proteins in order to facilitate localized gene expression and protein translation to promote cell migration. This hypothesis requires testing in future experiments.

Validation of proteins identified in the CLD fraction by methods such as immunocytochemistry is needed to conclude that a protein associates with CLDs. It is possible that some proteins identified localize near, but may not directly associate with, CLDs. CLDs interact with multiple cellular organelles (119) and proteins associated with an interacting organelle may be isolated with the CLD fraction. Since we have not validated all the proteins in our analysis for cellular location *via* another mechanism, only hypotheses about their localization and function in cancer progression can be made. Despite this limitation, our analysis has generated a novel list of proteins that can be studied in more detail in future experiments.

In summary, we characterized CLDs and the CLD proteome isolated from the human metastatic breast cancer cell line, MCF10CA1a. The identification of an interesting variety of proteins in the isolated CLD fraction reflects both similarities with CLDs in other cell types, as well as differences that may support a novel role of CLDs in cancer. It is possible that proteins associated with CLDs in metastatic cancer cells may play a role in permitting the advantageous metabolic plasticity that supports cancer progression. It would be interesting to assess the similarities and differences of CLD proteomes in other metastatic breast cancer cell lines which may further our understanding of cancer progression and identify factors that can be targeted to prevent metastasis. In conclusion, this study provides a new perspective on the role of CLDs in breast cancer metastasis.

## Data Availability Statement

Raw LC-MS/MS data is available on the Mass Spectrometry Interactive Virtual Environment (MassIVE) data repository at ftp://massive.ucsd.edu/MSV000086731/.

## Author Contributions

KB and DT conceived and designed the study. AZ and CA performed the experiments, analyzed the data, and wrote the first draft of the manuscript. All authors contributed to the article and approved the submitted version.

## Funding

This work was supported by the Purdue University Center for Cancer Research, Indiana Clinical Translational Science Institute NIH/NCRR Grant #TR000006, and the National Institutes of Health 5R01CA232589.

## Conflict of Interest

The authors declare that the research was conducted in the absence of any commercial or financial relationships that could be construed as a potential conflict of interest.

## Supplementary Material

Supplementary Figure 1 Representative Ponceau stain for Western blots. Fractions were loaded by volume: 10 μL floating fraction (FF)-10, 5 μL pellet (P) and whole cell lysate (WCL). Membrane demonstrates the relative amount of protein per lane.

Supplementary Figure 2 CLDs are not present in non-metastatic MCF10A-*ras* cells. Representative transmission electron microscopy image (TEM) of a MCF10A*-ras* cell, scale bar 5 μm.

Supplementary Table 1 LC-MS/MS results of all proteins identified.

Supplementary Table 2 Gene Ontology_Biological Process (GO_BP) enrichment. Sheet 1: GO_BP enrichment of identified proteins; Sheet 2: Individual identified proteins and their GO_BP terms.

## Abbreviations

ABHD5, 1-acylglycerol-3-phosphate O-acyltransferase ABHD5; ATGL, adipose triglyceride lipase; CANX, calnexin; CHO, Chinese Hamster Ovary; CLD, cytoplasmic lipid droplet; EMT, epithelial-mesenchymal transition; ER, endoplasmic reticulum; FF, floating fraction; GAPDH, glyceraldehyde 3-phosphate dehydrogenase; GO_BP, Gene Ontology Biological Process; GPAT4, glycerol-3-phosphate acyltransferase 4; HMGCS1, hydroxymethylglutaryl-CoA synthase; IQGAP1, IQ motif containing GTPase activating protein 1; LC-MS/MS, liquid chromatography-tandem mass spectrometry; LFQ, label-free quantification; MS, mass spectrometry; NSDHL, sterol-4-alpha-carboxylate 3-dehydrogenase; PBS, phosphate buffered saline; PCYT1A, choline-phosphate cytidylyltransferase A; PLIN, perilipin; PNPLA2, patatin-like phospholipase domain-containing protein 2; SDS-PAGE, sodium dodecyl sulfate-polyacrylamide gel electrophoresis; SQLE, squalene epoxidase; STK24, serine/threonine kinase 24; TAG, triacylglycerol; TEM, transmission electron microscopy; WCL, whole cell lysate.
