# Supplementary figures and images for: Proteomic Characterization of Cytoplasmic Lipid Droplets in Human Metastatic Breast Cancer Cells
Source: Front Oncol. 2021 Jun 1;11:576326. doi: 10.3389/fonc.2021.576326 (PMC8204105; doi:10.3389/fonc.2021.576326)

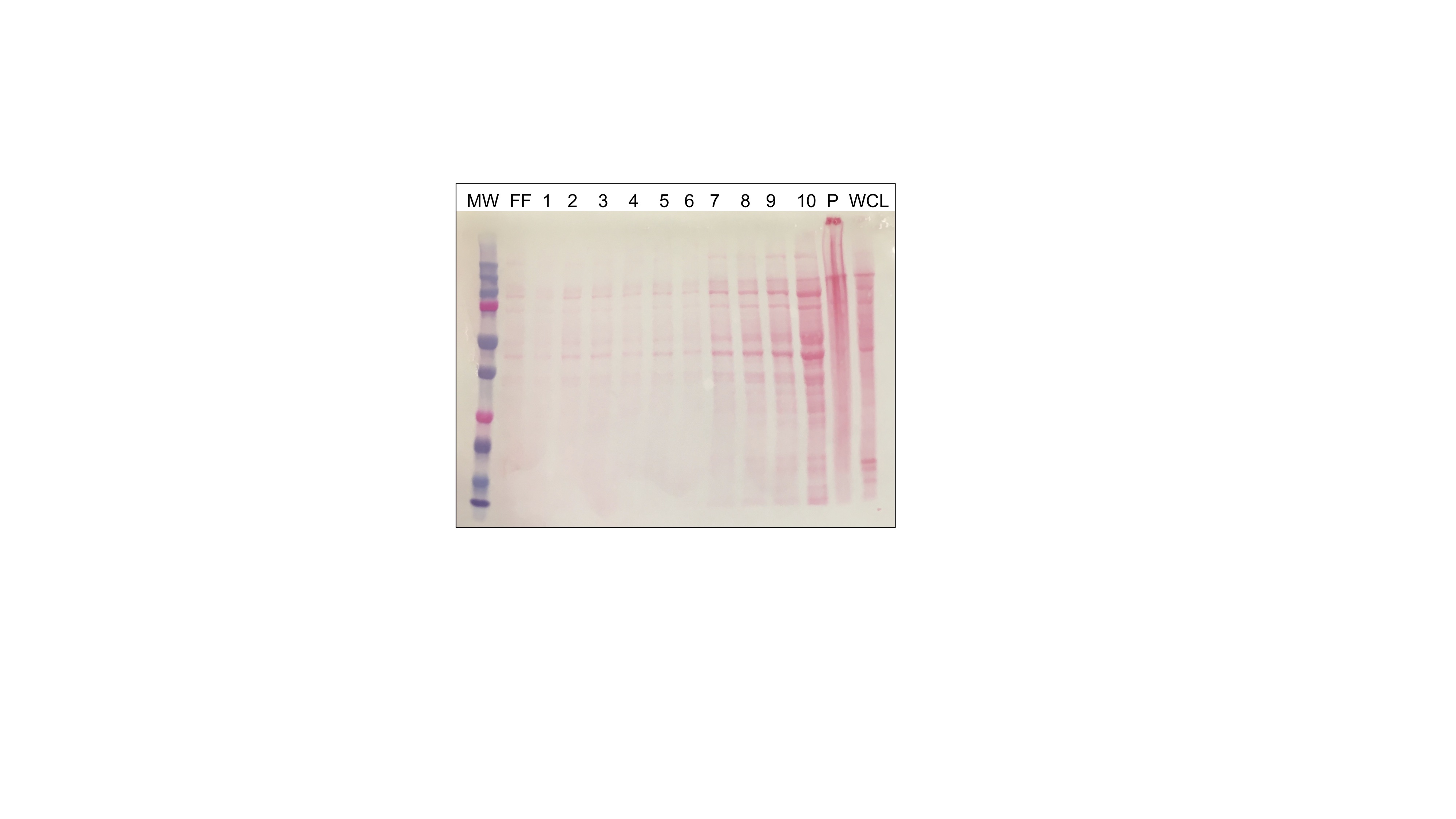

Supplement: Supplementary Figure 1 — Representative Ponceau stain for Western blots. Fractions were loaded by volume: 10 μL floating fraction (FF)-10, 5 μL pellet (P) and whole cell lysate (WCL). Membrane demonstrates the relative amount of protein per lane. [file Image_1.jpeg]

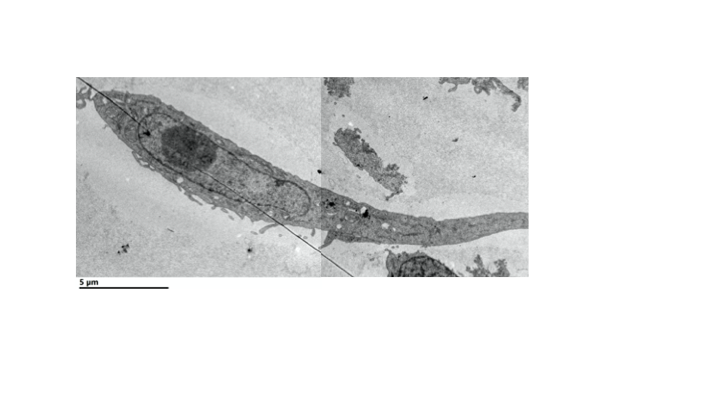

Supplement: Supplementary Figure 2 — CLDs are not present in non-metastatic MCF10A-ras cells. Representative transmission electron microscopy image (TEM) of a MCF10A-ras cell, scale bar 5 μm. [file Image_2.tiff]
